# Contours of Positive Mental Health: An Exploratory Delphi Study from India

**DOI:** 10.3390/bs9070066

**Published:** 2019-06-26

**Authors:** Diptarup Chowdhury, Ahalya Raguram, Seema Mehrotra

**Affiliations:** 1 Department of Clinical Psychology, Lokopriya Gopinath Bordoloi Regional Institute of Mental Health (LGBRIMH), Tezpur, Assam 784001, India; 2 Professor Emeritus, Department of Clinical Psychology, National Institute of Mental Health and Neurosciences (NIMHANS), Bangalore 560029, India; 3 Department of Clinical Psychology, National Institute of Mental Health and Neurosciences (NIMHANS), Bangalore 560029, India

**Keywords:** positive mental health, well-being, Delphi method, dimensions of well-being in India

## Abstract

The need to broaden attention from a narrow focus on mental illness and pathology to investing in understanding and enhancing positive mental health is well recognized. The bulk of research on positive mental health tends to be largely based onwestern developed nationconceptualizations. This highlights the need to examine the construct of positive mental health in diverse socio-cultural contexts. Present study aimed at exploring indicators and dimensions of positive mental health in Indian adults. A two-step Delphi technique, involving a vignette-based tool was used for facilitating a contextualised understanding of indicators of positive mental health. The sample consisted of a panel of 35 experts from different regions of India, majority of whom had >10 years of professional experience. Using a bottom-up approach, content analysis of the emergent data in terms of responses of experts to 10 vignettes resulted in identification and operationalisation of 33 indicators which could be grouped under 13 dimensions. Comparisons with popular well-being frameworks indicated that eight of these could be considered newer dimensions. Though the remaining dimensions significantly overlapped with existing dimensions of well-being in the western literature; finer differences in their meanings and constituents were observed. Finding have implications for further research.

## 1. Introduction

The World Health Organization (WHO) defines mental health as “a state of well-being in which the individual realizes his or her own abilities, can cope with the normal stresses of life, can work productively and fruitfully, and is able to make a contribution to his or her community” [1]. This is a positive conceptualization of mental health. Similarly, Keyes proposed that the conceptualization and measurement of mental health should include not only absence of mental illness but also the presence of positive features of mental health which in turn include emotional, psychological and social well-being [2]. There is in fact cumulative research evidence that dispels the assumption that “well-being would prevail when pathology is absent” [3]. All these observations reflect a growing interest in looking closely at the construct of positive mental health as distinct from mental illness [2,4].

## 2. Literature Review

### 2.1. Emotional, Psychological and Social Well-Being

The WHO definition cited above highlights well-being as a core aspect of mental health. Well-being is a popular term used in myriad ways across disciplines, and here we present a brief overview on the common usage of this term in the field of psychology. Emotional well-being (i.e., low frequency of negative emotions and high frequency of positive emotions), along with cognitive evaluations of life as satisfying also referred together as subjective well-being-are the most examined forms of well-being [5,6]. However, the notion of positive mental health does not merely comprise of emotional well-being or the ‘feeling good’ (hedonic) aspect. There is a consensus about the need to investigate constructs that may be indicative of high/positive psychological functioning, loosely termed as the ‘doing well’ (eudaimonic) aspect [7,8]. The latter is often operationalized in terms of psychological well-being. The most popular conceptualization of psychological well-being incorporates six dimensions namely, self-acceptance, positive relations with others, personal growth, purpose in life, environmental mastery and autonomy [9]. In addition, it has been argued that positive mental health needs to extend beyond the personal sphere to incorporate social well-being. Individuals high in social well-being perceive society as meaningful and possessing a growth potential, feel belonged to and accepted in their communities and see themselves as contributing to society in some way or another [10]. While the hedonic perspective argues for maximising of one’s pleasurable moments as the pathway to well-being; the eudaimonic perspective makes a case for leading a life of virtue and actualizing of one’s inherent potentials as the pathway to well-being [11]. Following this distinction, the major works of Diener [12], Kahneman [13], (hedonic well-being) and Seligman [14] (authentic happiness) can largely be placed under hedonic approach of well-being, while works of Waterman [15] (personal expressiveness), Ryff [9], Ryan & Deci [16] (self-determination theory), and Keyes [2] (positive mental health/flourishing) can be broadly placed under eudaimonic approach to well-being. There is burgeoning evidence-base on beneficial outcomes of investing in positive mental health/well-being from a public health perspective [2,4,17,18].

### 2.2. Limitations of Current Conceptualisations of Well-Being and Rationale for the Present Study

Notwithstanding the increased attention, the field of well-being is plagued with many challenges and unresolved issues. These challenges start with the very conceptualization of ‘well-being’ and the prevalence of multiple terminologies that may be ill-defined or used loosely [19]. Moreover, though several research-studies have investigated the constructs of life-satisfaction and emotional well-being—the ‘feeling good’ component of well-being; there are fewer empirical studies on examining positive functioning or eudaimonia—the ‘doing well’ component.

Despite the divergence of definitions and overlapping terminologies, Ryff’s model [9] of psychological well-being (PWB) remains the most-researched construct in this field. PWB and its six constituent-dimensions were arrived at by extracting core themes from existential, humanistic and developmental perspectives of Psychology—a discipline primarily rooted in Euro-American intellectual-soil. Inconsistent factor-structure of PWB, evident in multiple cross-cultural empirical studies, raises questions about the validity of PWB dimensions across various cultural-contexts [6,20,21]. Ryff and her colleagues attempted to operationally define the various dimensions of PWB and developed popular scales to measure them [9]. But the fundamental aspects of the dimensions (e.g., how these may be manifest in day-to-day lives in specific cultures) as well as the comprehensiveness of the same across cultures remains to be well understood. The salience of different constituents of positive mental health/well-being may vary from culture to culture. Empirical evidences have accumulated over time for the hypothesis that happiness and well-being are significantly grounded in socio-cultural modes of being a person and interacting with others [22]. Research in the field of well-being/positive mental health have been gaining significant attention, particularly in the developmental and health policy frameworks in developed nations, but it is argued that these studies largely follow a paradigm based on a specific kind of world-view, typically referred to as Western, Educated, Industrialized, Rich, & Democratic (W.E.I.R.D) and findings from such research whileoften labelled as ‘universal theories’ may be erroneously applied to other cultures [23,24].

Against this backdrop, the present study was aimed at developing a contextualized understanding of the dimensions that may constitute positive mental health in the contemporary Indian socio-cultural context. This was achieved through consultations with a panel of local experts with the help of a Delphi method and utilisation of vignettes to elicit expert views on salient indicators of positive mental health. This study formed part of a larger multi-phasic study that was carried out to develop and pilot test a culturally grounded interview—based measure of positive mental health.

### 2.3. Study Objective and Operational Definitions Used

The specific objective of the study was to develop a set of indicators of positive mental health and identify corresponding dimensions appropriate for Indian adults. The following operational definitions of the terms relevant to the study objective were used: a) Positive Mental Health (PMH): PMH is broadly understood as combination of both hedonic and eudemonic aspects of well-being [25]. In the present study, positive mental health was defined at the outset as a state that involves features of emotional, psychological and social well-being [4]. It was expected that the study findings would result in further refinement and operationalization of the state of positive mental health; b)Indicators of PMH: Specific markers of behaviours, thoughts or feelings or any combination thereof that reflect the on-going state of PMH and are identifiable/can be rated based on self-reports;c) Dimensions of Positive Mental Health: It was planned that indicators of PMH that would emerge in the study would be grouped together based on shared features to form the dimensions of PMH. Their similarities with the existing dimensions of psychological well-being as well as social well-being as given by Ryff [9,26] and Keyes [10] were examined during the study.

## 3. Method

The study objective was achieved through the use of through a two-step Delphi technique involving a panel of experts.

### 3.1. Rationale on the Method Adopted:

A culturally contextualized route to arrive at relevant dimensions of positive mental health is through identifying the salient indicators (of thoughts-actions-feelings) of positive mental health. Such an indicator-centric process is essentially a bottom-up process, grounded in context-sensitive data. Vignettes are considered simulations of real life scenarios. A Delphi survey-format which uses vignettes, can be useful to elicit research-participants’ knowledge, attitude or opinions as to what may indicate positive mental health in the case of a given person depicted in a given vignette. Hence it was decided to involve a heterogeneous pool of experts from India as participants and employ vignettes as the basic context to generate PMH indicators. The abstractness of the various concepts related to eudaimonic well-being literature can make their operationalization a challenging exercise. Hence, anchoring on ‘indicators’ of well-being that are experience-derived, concrete and specific, could be a way to minimise such limitations.

The experts, while responding to the culturally-sensitive vignettes, could contribute to generating a pool of indicators based on their observations and experiential knowledge of how individuals maintain and enhance their well-being in day-to-day life. It was expected that an attempt at consensus-building among the experts, typical of the Delphi research method would result in finalising the indicators through two rounds of consultation. Emergent dimensions, made up of constituent-indicators, could then be compared with the existing dimensions of well-being by Ryff and others to arrive at a clearer and more contextually-grounded understanding of dimensions of positive mental health.

The Delphi techniquehas been described as ‘how to hold a meeting when there’s no one there’ in that it employs a postal-mail/e-mail survey methodology to determine the range of opinions of ‘experts’ (called panelists) on particular matters and to explore (or achieve) consensus on disputed or less-researched topics [27]. It provides an “efficient and effective way to involve busy experts who may not be able to come together to interact with each other to investigate theoretical and practical issues and ultimately build consensus” [28]. It is often conducted across a series of two or more sequential survey questionnaires known as ‘rounds’ where ideas/responses of initial round are collated and a detailed quantitative and/or qualitative feedback is provided to the panelists to elicit their subsequent ‘revised’ responses. Although there are variations in the number of rounds and the size of the panel in Delphi method, depending on the nature of the exercise as well as feasibility, 2–3 rounds may be sufficient to generate adequate data. With regard to the number of panelists, it is recommended to have at least 10 experts [29]. This technique was considered appropriate for the present study for the following reasons: ability to help in arriving at culturally relevant indicators of PMH through generating viewpoints of researchers and practitioners in the country; scope for drawing together the existing limited knowledge on specific indicators of PMH and highlighting areas of agreement/disagreement; enabling a group communication that otherwise might have been impossible due to geography, time or other constraints. Moreover, anonymity between panelists can encourage creativity, honesty and balanced consideration of generated ideas/opinions.

### 3.2. Procedure at a Glance

A panel of experts (also called Delphi-panelists) was enlisted. Two rounds of Delphi survey were conducted with this panel. In the first-round, they responded to a vignette-based survey tool (VBST) and a list of potential PMH indicators and dimensions were generated. In the 2nd round of survey, this list was refined and an attempt was made to generate consensus among panelists.

### 3.3. Sampling Strategy

Purposive sampling was used to recruit thepanelists in this study. As research in this area has been scarce, a broad definition of ‘experts’ was adopted which consisted ofmental health professionals, as well as other related professionalsincluding psychologists, psychotherapists, counselling psychologists andbehavioural science trainers, who had been working in the field of mental health and well-being, either at the level of research and training or client-service for at least 5 years. An attempt was made to reach out to a heterogeneous sample of experts from across the country using email/postal correspondence and follow-up telephonic contacts, if needed. Diversity of this sample was attempted in terms of getting experts with varied: (a) years of experience, (b) nature/settings of work, (c) geographical regions, and (d) interest in the field of positive mental health/well-being as indicated through their involvement in preventive and promotive programs and research. The samplesize for Phase I was 35 Delphi survey panelists. The study was initiated after obtaining ethical clearance from the committee which reviewed the proposal in the researchers’ department and their institute’s Ethics Committee.

### 3.4. Tools

#### 3.4.1. Tools Used in Phase I (Delphi Survey)

1. Basic Data Sheet: This was developed for the study to record basic information about the panellists in Delphi survey and included socio-demographic details, and professional background.
2. Vignette-based Tool (VBT): This tool was specifically developed to elicit potential indicators of PMH for use during Delphi survey with experts. A total of 30 short vignettes were initially created by the researchers (during pilot-work) which were later shortlisted to 10. These depicted hypothetical individuals varying in terms of developmental phases in adulthood (age), gender, marital status, educational level, and being in varying life circumstances. The participants (experts) were asked to respond to the vignettes by generating what might be specific indicators of positive mental health in the case of the hypothetical persons depicted in the respective vignettes. They were provided with the operational definition of indicators as mentioned earlier.
3. The key purpose of using these vignettes for Delphi—survey was to help the experts to come up with culturally relevant indicators of positive mental health-not in abstraction, but in the background of multiple life- contexts embedded across vignettes. Use of vignettes was also expected to help in allowing the emergent indicators to be closely linked to the manifestations of positive mental health in day-to-day life of individuals. A brief background of the study, and guidelines along with examples were added as prelude to the vignettes. Care was taken to develop the vignettes in a culturally-sensitive manner. A brief pilot-study was carried out using consultation with a few experts (N = 5). This step was meant to develop the final version of this survey-tool which had a total of 10 vignettes.

#### 3.4.2. Delphi Survey Proforma (for Round-2):

This proforma was created at the end of 1st round of the Delphi-survey to serve dual purposes: (a) To provide panelists a quick glimpse of the results of round-1 (through summary-tables), (b) to elicit their comments/responses in order to deepen the understanding of PMH-indicators/dimensions and their inter-relations. Use of Delphi in the field of well-being research, though infrequent, has also been tried out by other researchers [28]. The literature alsorecommends the Delphi technique as a part of research design, geared towards exploration of construct(s) and theory-building [30,31].

### 3.5. Detailed Procedure

#### 3.5.1. Step-1 Development of Vignette Based Tool

A total of 30 short vignettes were initially created by the researchers which were later shortlisted to 20. Each of them depicted a hypothetical individual with varying age (within 20–50 year range), gender, marital status, educational level, and life circumstances. Equal numbers of individuals represented each gender (i.e., 10 males and 10 females). They varied in terms of marital/conjugal status (viz., single, married with/without children, widow, divorcee, live-in relationships), sexual orientation (e.g., being a gay), educational level (primary/lower-levels of education to highly educated/professional qualification), working status (e.g., home-maker, student, self-employed, unemployed, house-maid, farm-labourer, salaried persons etc.), and life-circumstances/stressors (e.g., daily-hassles and role-conflicts/strains, chronic physical health problem, decision-making challenges etc.). On the whole, care was taken to create vignettes with wide-ranging socio-economic-educational background to represent the diversity of Indian adult population (20–50 yrs) to the extent possible. These varied vignettes formed the contexts in which the expert-participants generated specific indicators of PMH.

To refine the vignettes five senior professionals from the fields of Clinical Psychology, Psychiatry/Behavioural Sciences were recruited as experts for the pilot study. Purposive sampling was used to recruit this sample with an attempt to ensure that these professionals had at least 10 years of professional experience and a significant part of their work involved a focus on well-being. In line with this, the sample consisted of professionals whose experience ranged between12 and 25 years, with a consistent focus on areas of well-being/positive mental health in their professional work. The expert-participants read each of the 20 vignettes and listed out the possible PMH indicators for each of the hypothetical persons. The PMH indicators, in this context, were operationally defined as the pattern of feeling-thinking-doing (in the hypothetical person) that would indicate high/positive psychosocial functioning at that point in her/his life.

After organizing the responses received from five expert-participants across 20 vignettes, the responses (words, phrases, sentences, paragraphs) were read carefully and key descriptions denoting positive psychological functioning or positive mental health as mentioned in the context of various vignettes were identified. Looking at the key phrases, codes/labels were developed which conveyed the essential description of a particular PMH state. These codes represented the PMH-indicators. The process of coding was done independently by two of the authors (DC & SM) and the positive mental health dimensions (broader categories) were arrived at after resolving differences through consensus. The initial findings from this pilot phase were reviewed to see the extent of mapping of the pilot-data onto the already-established psychological and social well-being dimensions proposed by Ryff [9] and Keyes [2]. The major objective at this preliminary phase was to confirm whether: (a) the vignettes were, in reality, able to elicit specific indicators/markers of PMH, (b) findings were consistent with already existing literature, and (c) potentiality of vignettes to generate newer indicators/dimensions, beyond that of Ryff’s/Keyes’s frameworks. It was observed that the vignettes yielded indicators which could be subsumed under five of the six dimensions of Ryff. Environmental mastery, positive relations, autonomy, purpose in life, personal growth. Life satisfaction which is an established subjective well-being dimension also emerged in the pilot phase. In addition regulating emotions (with an emphasis on acceptance), maintaining positive future orientation, meaningful engagement and self-care emerged as newer dimensions which are not part of Ryff’s psychological well-being dimensions.

After analyses of pilot data VBT (trial version) and general feedback from the pilot expert-participants, a need for shortening the survey-tool was felt. All the vignettes and the elicited responses (indicators) were reviewed and 10 vignettes (out of 20) were retained in the final version of VBT. Here are two examples from the final set of 10 vignettes: a) “F is a 29 years old commerce graduate who comes from a conservative, large family. He has been working in the family business since he passed out from college. Though he has had many friends from both the genders; he recently realized that he is gay. He is being pressured to marry a girl who has been chosen by his family.” b) “A is a 47 years old woman working as a telephone operator in a government organization. She lost her husband in a road traffic accident about one year ago. Her only daughter got married 5 years ago and stays with her in-laws and husband in another city. The daughter contacts her over phone about once in a week and visits once every 4–6 months.”

The following criteria were used in shortlisting the vignettes: wide coverage of multiple dimensions (both Ryff’s and potentially new); more or less equal representation of vignettes based on socio-demographic/life-circumstances variables; judicious selection of vignettes which were found to be saturated with some of the frequently-occurring dimensions/indicators (e.g., Environmental Mastery, Positive Relations); general feedback of the expert-participants about the vignettes with respect to their suitability for the socio-cultural context. These 10 vignettes were found to be potentially capable to elicit both Ryff’s dimensions (of psychological well-being) and potential ‘new’ dimensions. Thus the final version of VBT was found to be suitable to be used for the round-1 of Delphi-survey.

#### 3.5.2. Step-2 Administration of Vignette-Based Survey Tool (VBT) Delphi Round1

Fifty potential expert-participants from various parts of India were identified and were contacted over email/phone soliciting their participation. Ten of them did not respond back within the pre-determined time-frame (approximately 2–3 weeks after initial contact-email). Five others declined due to time-constraints. Finally, 35 expert-participants who agreed to participate were sent the informed consent form over email and/or postal mail and were requested to give their explicit consent via email/postal mail. Once their informed consents were received, VBST was mailed to them.

#### 3.5.3. Step-3: Synthesis of the Responses Elicited through Vignette-Based Survey Tool

A bottom-up approach was followed (data (responses) => indicators => dimensions). It was planned to collate data collected through vignettes and to collapse indicators which were synonymous/highly overlapping. Subsequently, probable dimensions (broader categories) were identified by grouping of the indicators by the researchers, based on data of Delphi Round1 as well as the available literature. Care was taken to first identify the indicators in a contextually grounded way (i.e., from responses of experts elicited through vignettes) and thereafter grouping of the indicators was carried out based on their conceptual similarities. Thus, broader dimensions were identified, corresponding to the indicators already generated. This step provided an opportunity to assess conceptual validation and relevance of the existing model/framework(s) of PMH in the contemporary Indian context.

#### 3.5.4. Step-4: Eliciting Feedback on Preliminary List of Indicators and Corresponding Dimensions: Delphi Round2

It was planned to circulate the refined list of indicators and proposed dimensions, along with their descriptions, to all the panelists in the 2nd round of the Delphisurvey. Their feedback/opinion on the indicators and proposed dimensions were collected through a survey proforma (viz., Delphi Survey ProformaRound 2).

#### 3.5.5. Step-5: Finalization of the Indicators and Their Corresponding Dimensions

After receiving the feedback/fine-tuned responses from the 30 out of 35 experts within the predetermined time-frame (drop-out: N = 5), the indictors and their corresponding dimensions were finalized through discussions within the team of researchers.

## 4. Results

### 4.1. Sample Characteristics

The expert-panelists involved in the present study were a diverse group of people having a wide range of age, location, and salient personal and professional characteristics. Panelists were found to have a variety of primary work-responsibilities, several were playing multiple professional roles. They represented nine different cities from the northern, southern, western and eastern regions of India. Their educational background and expertise were also fairly varied in terms of their qualifications, subject-areas and work-settings.A sizable proportion of the sampled participants were from the field of mental health. It was expected that their basic grounding in mental health, coupled with practice- experiences/insights based on interactions with clients and their caregivers in varied (often challenging) contexts would be of use in picking up indicators/nuances of psychosocial functioning. However, even within mental health, there was diversity in terms of professions- ranging from clinical psychology, psychiatric social work to psychiatry. In addition, 26% of the participants were professionals from other more diverse backgrounds involved in teaching or training activities/research in areas related to well-being/positive mental health. (Table 1 and Table 2).

All 35 expert-participants responded to each of the 10 vignettes. Each vignette generated, on an average, approximately four responses (words/phrases/sentences/occasionally paragraphs) per participant, close to 1400 data-units were available for analysis. They were read carefully and key descriptions denoting positive psychological functioning or positive mental health as mentioned in the context of various vignettes were identified.Looking at the key phrases, codes/labels were developed (viz., PMH indicator) which conveyed the essential description of a particular PMH state. Synonymous/overlapping codes were collapsed with each other and distinct codes were retained at the end of the qualitative (content) analyses.Subsequently, probable dimensions were identified (from the indicators), based on data of Delphi round-1 as well as the available literature The whole process was done independently by the first and third author and the final list of 33 PMH-indicators (codes) and their descriptions were arrived at after resolving differences through consensus.

### 4.2. Findings from Delphi Survey (Round-1)

#### 4.2.1. Positive Mental Health Indicators

A large number of indicators (total 33) emerged from the content analysis of Delphi Round-1 data. (Table 3) It may be noted that as far as quantitative analysis is concerned, only descriptive statistics (frequencies and percentages) have been used. The descriptions of the indicators mentioned above were used for communicating to all the expert-participants in the next round of Delphi-survey.

In the next stage, the codes (PMH indicators) were reviewed and were grouped into (broader) categories, called dimensions of PMH. Again, the whole process was done independently by the first and the third author and a list of 13 PMH-dimensions and their descriptions (definition) were arrived at after resolving differences through consensus and discussions amongst all three researchers.

#### 4.2.2. Positive Mental Health Dimensions

Although the dimensions were often represented by multiple indicators, some of the indicators themselves were retained as dimensions (viz., Acceptance of reality, Self-care, Meaningful engagement, Meaning-making, and Contentment & gratitude). The indicator ‘Dialogue for conciliations’ was put under two dimensions: ‘Mastery & Competence’ and ‘Positive Relations with others’, as content analyses at this stage, provided indications for both (Table 4).

Some of the indicators were found to occur very frequently across vignettes and across expert-panelists (e.g., mobilizing support, acceptance of reality, etc.). A few other indicators, on the other hand, were found to occur less frequently (e.g., following norms, applying strengths, etc.) (Table 5).

### 4.3. Findings from Delphi Survey (Round2)

As part of Delphi Round2, the list of PMH indicators and proposed dimensions, along with their definitions, were circulated amongst the Delphi-panelists. A summary of quantitative findings of Delphi round-1 was also provided to them as feedback. They were asked to express their degree of endorsement towards the proposed dimensions and the indicators and their inter-relationships as well. Five out of the 35 panelists did not respond within the prescribed time-frame during Round2 for personal other unanticipated extraneous reasons. Thus data from 30 panelists were used for analyses in Delphi Round2. Table 6 depicts the degree of agreement among expert-participants regarding match between PMH-indicators and the over-arching dimensions.

At least 3/4 of the panelists agreed with the grouping of indicators under the dimensions to which these were assigned by the researchers based on Round1 data. (Table 6). Divergences of opinions were evident in case of three PMH indicators namely,‘dialogue for conciliation’,‘following norms’, and ‘courage’. Thefirst round data had thrown up the possibility of grouping dialogue for conciliation under two dimensions- mastery &competence (M&C) and positive relations with others (PR). Hence opinions were solicited from the panelists in Round2 about it. All of them (100%) agreed to have this subsumed under PR.Moreover, a significant proportion (40%) of the sample was emphatic in that it was not appropriate at all to group it under M&C. Hence, ‘dialogue for conciliation’, as a PMH-indicator was finally retained only under PR dimension. Similarly, opinions were solicited on the indicator ‘following norms’in Round 2 of the Delphi survey. Only five panelists mentioned that it candefinitely be treated as a PMH indicator, with some of them considering it as a confidence-booster. Example: “Conformity to existing norms gives one self—confidence on the basis of being approved in the context.”Several others opined that ‘following norms’ may or may not be an indicator in a given context, depending on multiple factors.A few examples of this stance are as follows:*“this may be a PMH indicator when accompanied by self-reflection & clarity/self-acceptance/personal growth”;”it may be a means of reducing conflict and getting social advantage, without compromising on honesty”; “it is associated with ambiguity as norms themselves are relative”*; ‘Following norms is an indicator of positive mental health, only if it indicates a positive acceptance of reality and is not accompanied by a dip in self-esteem and future resentment”. There were a few others who expressed that ‘following norms’ is clearly not a PMH indicator: They opined thatgiving up one’s valued goal gives rise to internal/unresolved conflict or that it does not denote environmental mastery, rather the reverse. Rejecting this as an indicator of PMH, one of the panellists stated that it entails a *“commonly practiced way to control a person’s individuality and freedom and thereby results in loss of PMH”;”I don’t agree that “following norms” is always a positive mental health indicator. I think being able to follow one’s true self is very important, and in situations where one’s true self is in conflict with societal norms, following societal norms might not always be the best thing to do. The person would probably go through pain in either choice they make, and would have to grieve the loss (whether it is the loss of societal approval or loss of personal desire), but sometimes, not following the norms might eventually bring more positivity to the person”*. Courage was another PMH indicator, wherein differences of opinions were noted. Half of the panelists agreed completely with this indicator’s grouping by the researchers under the autonomy-relatedness dimension:*“Courage is a part of autonomy or responsibility”*. However the other half suggested either regrouping or redefining of this indicator. Over 1/3rd of the panelists suggested it to be either regrouped under Self-reflection & Clarity or M&C or to separate it out completely as an exclusive dimension by itself. A few also suggested redefining it. For example:*“…The word ‘courage’ seems misleading. If as you say, it is related to autonomy-dependence – why not look for a better term so that it fits here? ”or “I think ‘courage’ is an important PMH dimension in itself. However, the definition would be broader than the one given under autonomy-relatedness. It would include the capacity to take risks to achieve valued goals, to venture out of familiar, comfort zones, to experiment, to be open to new experience and learning. In this sense it would have bearing on the personal growth dimension…”*

A majority (3/4 or more) of the panelists found all the new dimensions to be relevant (Table 7). The new dimensions were not already well-established ones as per the existing well-being frame-work/literature, especially work done by Ryff and her colleagues. Among these eight new dimensions, the most endorsed ones were ‘Self-reflection & Clarity’ (90%) and ‘Emotional regulation’ (90%). Examples of a few comments/responses of Delphi-panel (Round 2) endorsing their relevance: *“…especially for women who are in traditional culture…”* (a panelist’s comment regarding Self-care); *“…In general I feel the additional dimensions provide a wider framework by incorporating the Eastern viewpoint on the human personality…”* (a panelist’s comment regarding all new dimensions, in general).

The panelists were also asked to list out a combination of top-5 PMH-dimensions (out of 13) which may be considered critical in shifting somebody’s psycho-social functioning from average to above-average. Twenty five panelists responded to this item in the survey.

As evident from Table 8, almost 3/4 of the panellists (72%) endorsed M&C as the top-most critical dimension. The experts also commented on the interrelationships amongst the dimensions. *“…I would put self-reflection and clarity first, combined with an acceptance of reality, which should ideally include acceptance of the people around as well as the environment. Once you know what/who you are, and can accept (and understand and empathise with) the people around you, you next need emotional regulation to allow you to put your plans in action. It will then be easier to move towards mastery and good relationships, which (I hope) will lead to meaningful engagement…”; “…In my opinion, the dimensions of mastery & competence, positive relationships, emotional regulation, acceptance of reality, self-acceptance & self-care relate to average positive functioning. The rest of the dimensions—self-reflection, meaning-making, meaningful engagement, personal growth, autonomy relatedness, giving, contentment & gratitude- describe above-average positive functioning. However, my sense is that the PMH dimensions in the first group are a necessary condition without which those in the second (above average functioning) category may not have solid foundation…”*.

## 5. Discussion

### 5.1. Observations on Utility of the Delphi Method for the Study

The development of the vignettes was of utmost importance for the present study as it contained the essential ingredients for subsequent generation of PMH indicators. Unlike ‘quantitative vignettes’ (vignettes employed within a quantitative approach, generally integrated within a large-scale questionnaire and responses elicited using a Likert-style format), ‘qualitative vignettes’ are utilized differently. By virtue of their flexibility in content and format, these vignettes facilitate explorations of meanings and interpretations not easily accessible through other methods [32]. The vignettes embodied hypothetical individuals. Brief slices of lives of 20 such individuals were created depicting a wide range of socio-demographics and life-circumstances that would typically be witnessed in Indian adults across the age range of 20 to 50 years. Thus, a certain degree of match was achieved between the vignette characteristics to the target participant-group about whom the present study’s discourse revolves around. The length of the vignettes was kept limited, thus ensuring that the content of each remains parsimonious. The characterization of each of the hypothetical individuals involved only two aspects: (a) particular socio-demographic context of the individual, and (b) the specific life-situation the individual is currently dealing with. The vignettes themselves did not provide any cues of possible solutions or ways of dealing with the situation at hand. This is in line with other researchers who have widely used vignettes and the typical recommendation that ‘vignettes should provide enough contextual information for respondents to clearly understand the situation being portrayed, but be ambiguous enough to ensure that multiple solutions exist’ [33,34,35]. Each of the vignettes was carefully worded, so as to avoid influencing the respondent’s answer: social desirability bias—a common pitfall in vignette-based researches [36]. The tool not only had a series of vignettes, it also had specific instructions and guidelines that ensured some parity of all respondents vis-a-vis background knowledge of the study. The piloting of these vignettes suggested their potential to generate new indicators/dimensions beyond the existing ones found in the literature and provided leads for developing a culturally-contextualized set of indicators and their corresponding dimensions of positive mental health.

### 5.2. Reflections on Characteristics of the Delphi-Survey Participants

The sample size (number of panelists) for this phase of research was satisfactorily large, as the approximate size of Delphipanels is generally under 50 [37]. A majority of Delphi studies have used 15 to 20 respondents [38]. However, there are no set standard for all kinds of Delphiresearch and study samples vary depending on the objective(s) of the research. Generally, a higher number of panelists are recommended for studies which enlist fairly heterogeneous samples, like the case in present study [39]. The attrition rate of panelists in the second round of Delphi was 17% (i.e., only five experts), a small number as per the general trends in Delphiresearch [40]. The panelists belonged to diverse age-groups, ranging from 32 years to 76 years. A large number of the participants (60%) were senior professionals. Most had been working in their own professional fields for a fairly long time (21 years or more) A broad definition of ‘expert’ was employed in order to recruit the sample and the group was fairly heterogeneous in terms of their educational qualification, specializations, current work-settings and primary work-responsibilities, though all had some expertise and special interest in the field of well-being. Such a heterogeneous group could be considered an asset for the present-study. The area of PMH or well-being is a broad-focused multidisciplinary one which can potentially be nurtured by professionals belonging to various related disciplines (viz., Clinical Psychology, Psychiatry, Psychiatric Social Work, Psychiatric Nursing, Psychoanalysis, Counselling Psychology, as well as Behavioural Science/Organizational Behaviour and other branches of Psychology). Three-fourths of the Delphi-panelists were mental health professionals. The panelists drew their expertise from varied experiences of working in different settings (hospital, private practice, teaching institute, industry, etc.) and also by remaining engaged with a diversity of professional-responsibilities in their day-to-day work. Previous studies have also found such heterogeneity in Delphi-panelists as a strength owing to the combined knowledge and expertise reflecting the full range/scope of the response-domain [41]. The Delphi-panel’s gender distribution was skewed; almost 2/3 of them were females. This may be accounted for the fact that more women enter the field of psychology, especially mental health. The under-representation of men in psychology and allied disciplines has been found to be a consistent trend in western countries [42]. India also mimics the same trend [43].

### 5.3. Generating PMH Indicators

Content-analyses of the responses resulted in 33 PMH indicators that the two researchers independently arrived at. Consensus within the research team was arrived at through discussions on minor discrepancies/disagreements in coding. This process of consensual validation of coding is a common practice in qualitative research to enhance the ‘quality’ of the qualitative analyses [44]. The indicators derived through the use of vignettes were contextually sensitive and grounded in experiences of day-to-day living, and not merely theoretically/conceptually driven. All the indicators generated prima-facie fulfilled the characteristics of PMH-indicators, as operationally defined. The indicators were all found to denote: (i) positive psychosocial functioning, (ii) internal subjective states of individuals, (iii) state-variables which are malleable to a large extent, and (iv) specific and clearly delineated constructs (variables) which are not ambiguous.

There was a fairly wide variation among the PMH indicators with respect to their spread (frequency of occurrence) and nature (content). Indicators were defined as high-frequency ones if they were generated in a majority (80% or at least eight out of total 10) of the vignettes, by a majority (80% or at least 28 out of the 35) of the panelists. Following this criterion, five indicators were found to be high-frequency indicators (viz., Mobilizing support, acceptance of reality, dialogue for conciliation, being flexible and openness to multiple perspectives, and sense of competency and mastery). Four indicators were found to be low-frequency indicators (viz., following norms, applying strengths, balancing needs of self and others, and accepting consequences of decisions/taking responsibilities). These were generated only in a few (less than 50% or four or less out of 10) of the vignettes, by a minority (30% or 10 or less out of 35) of the panelists. The remaining 24 indicators were spread in between these two groups, with moderate-to-high degree of occurrence.

It was speculated that the top-5 PMH indicators commonly occur in the context of challenges faced by individuals in their day-to-day lives. These are probably the typical contexts where they need to engage with others and/or respond to situational demands skilfully. Majority of the vignettes in the VBT depicted hypothetical persons in various challenging situations and thus possibly generated these five PMH indicators more frequently.

Responses generated in the Delphi Round1 also helped in developing descriptions of all the 33 indicators. The descriptions were fairly elaborate. The elaborateness of the descriptions can be considered as a methodological strength as it captures the breadth of the content of the particular PMH indicator. For example, ‘Dialogue for conciliation’, one of the frequently occurring PMH indicators, contains six defining characteristics in its description viz.: (i) it is a process of conflict resolution; (ii) it happens through communicating with significant other person(s); (iii) its goal is to arrive at agreeable solutions; (iv) it is achieved by means of decreasing resistances from others involved; (v) the process requires efforts in terms of dialoguing with others; (vi) the relevant other’s support is apparently unavailable.

### 5.4. Dimensions of Positive Mental Health

The elaborate description of the indicators that were already available facilitated the process of conceptually grouping together of indicators into broader dimensions. A total of 13 different dimensions emerged from the 33 indicators. Out of 13, five dimensions (viz., mastery & competence, positive relations with others, personal growth, autonomy-relatedness, and self-acceptance & respect) bear similarity with Ryff’s five PWB dimensions. Since conceptual similarity was the criteria for grouping indicators into dimensions, a few dimensions turned out to have multiple indicators subsumed under them. For a few, the conceptual overlaps seemed minimal and hence these were retained as single-indicator dimensions. Seven such single-indicator dimensions emerged, whereas, the remaining six dimensions had multiple indicators constituting them.

Two dimensions stood out as the broadest dimension viz., Mastery & Competence (10 indicators) and Positive Relations with others (six indicators). These two dimensions were very similar to Ryff’s two PWB dimensions viz., Environmental mastery and Positive Relations with others and are also replicated in other research studies as important dimensions of PWB [9,45,46,47]. ‘Mastery & Competence’ (M&C) is one of major multi-indicator dimensions that emerged in the present study. It is characterized by: (a) a sense of being in charge/control of one’s overall life-situation, (b) a sense of balancing multiple domains of life, (c) being flexible and accommodative of changes in plans/goals, and (d) being open to multiple options. All these indicators of M&C are geared towards managing demands in one’s life/environment for accomplishing valued goals/outcomes and maintaining overall sense of competence in life. Almost all the existing major theoretical/assessment frameworks include ‘Competence’ as one of the essential components of well-being/flourishing, representing aspects of perceived competence and self-efficacy [3,48]. Seligman [49] includes aspects of this dimension in his PERMA model as ‘accomplishment’. In all these frameworks, competence as a well-being component has been conceptualized in narrower terms, compared to how it has been defined in the present study. Ryff’s operational definition of ‘Environmental Mastery’ comes close to M&C with respect to its coverage [9]. However, her definition conveys a more outwardly-directed sense of mastery and competence (managing environment, controlling of complex array of external activities, effectively using surrounding opportunities; as compared to M&C in the present study. Mastery and competence in the present study included more of inward-directed efforts towards achieving a sense of control and balance by being flexible and open.

‘Positive relations with others’ (PR) is another major multi-indicator dimension in the present study that had multiple constituent indicators and thus represents a wide array of components. They ranged from investing in and maintaining relationships by taking recourse to multiple strategies (like emotional disclosures, dialoguing with others, showing sensitivity to others needs, supporting others as well as seeking support), to handling differences/conflicts sensitively in relationships. All other frameworks of well-being/flourishing include this component/dimension of well-being (i.e., positive relations with others). This universality of significance assigned to interpersonal well-being can be explained by Self Determination Theory which asserts that “people whose basic psychological needs for autonomy, competence, and relatedness are more satisfied experience greater wellness and thriving” [50,51]. The substantial emphasis on positive relationships with others in the present study indicates the salience of interpersonal well-being in the context of India, compared to other western countries. It could possibly be explained by the highly relational interdependent self-construal by the Indians where they try to define themselves in terms of their close relationships with others [52].

‘The third multi-indicator dimension of positive mental health generated in the present study is ‘Autonomy-Relatedness’ (A-R). This dimension consists of two aspects viz., expression of divergent opinions and independence in decision-making and action- while simultaneously attempting to actively maintain/nurture relationships with significant others despite disagreements. It is the presence of both these apparently contradictory elements (autonomy and relatedness) functioning conjointly, that makes this dimension unique and different from similar other dimensions/indicators mentioned in well-being frameworks in the West. Seligman [49] and Diener et al. [48] have not accorded a place to ‘Autonomy’ as a separate dimension/indicator of flourishing in their frameworks. It may be possibly due to the implicit presence of the notion of autonomy in conjunction with other indicators like competence. Huppert and So [3] and Ryff [9] have, however, placed ‘Autonomy’ as an important indicator/dimension of well-being. Nevertheless, it is self-determination and independence which has been emphasized in the western literature in the context of autonomy. ‘Autonomy-Relatedness’ as a well-being dimension in the present study, makes a significant departure in conceptualizing well-being through a different understanding of autonomy. This conceptualization of autonomy hyphenated (-) with relatedness (i.e., autonomy-relatedness) has been found by a few other well-being researchers [53]. Drawing evidence from Bangladesh, Devine, Camfield, and Gough argued that “coherent accounts of autonomy must always recognize the interdependence of people in groups, and that autonomy can coexist with substantial relationships of dependence” [54].

‘Emotional Regulation’ (ER) is another multi-indicator dimension generated from the present study which emphasizes aspects of both managing negative emotions and, experiencing and savouring positive emotions. The conceptualization about this dimension in the present study emphasized a contextual and dynamic nature of the ‘experience’ of emotional regulation rather than viewing it as a fixed and invariant trait. Existing well-being/flourishing frameworks include aspects of positive affect/emotion [2,3,48,49]. Huppert and So [3] in addition, also employed an indicator called ‘Emotional stability’ to assess flourishing, which is conceptually similar to ER, generated in the present study. However, ER, in the present study, appears to be the only dimension among the existing frameworks which seems to emphasize the aspect of ‘regulation’ covering both positive and negative emotions. Some studies have found robust cultural difference in emotional regulation where more interdependent cultures (like Asian/South Asian countries) were found to have stronger preferences to regulate their emotions, compared to Western countries which are more independent cultures [55].

‘Personal growth’ (PG), a uni-indicator dimension in the present study, features in Ryff’s (1989a) framework of well-being. The concept of PG in the present study appears to be circumscribed, emphasizing on awareness and goal-setting towards bringing about desired changes in personal attributes. Personal growth, as per Ryff’s definition, goes beyond the aspect of personal development, and emphasizes on self-realization and openness to new experiences [9]. In the present study, these two aspects of well-being are already included as components of Self-reflection & Clarity and Mastery & Competence, respectively.

‘Self-reflection and clarity’ (SR-C) comprised of internal clarity about oneself and one’s values as well as active review of life-goals and purpose, particularly at important points of time in one’s life. It does not have any direct corresponding dimension/indicator in any of the other existing well-being/flourishing frame-work. The emergence of dimensions such as self-reflection and clarity and acceptance of reality are in tune with the value placed on introspection and reflection on internal and external realities in the traditional Indian thought [56].

Another dimension generated in the present study viz., ‘Giving (social contribution)’ conceptually overlaps with the social contribution dimension/indicator in existing frameworks [2,3,48]. It comprised of themes related to doing one’s best in all that one does (‘giving’ of one’s best) as well as engaging in social causes/contributing to society in some way or the other.

‘Contentment & Gratitude’ is another dimension which was generated in the present study. Though all the existing frameworks have a dimension/indicator which represents positive emotions/feelings in general (includes wide range of positive affect), it is specifically contentment and gratitude that turned out to be salient well-being dimensions in present study. Both contentment and gratitude are in contrast to high-arousal emotions like excitement- valued highly in western cultures than its eastern counterparts [57]. Moreover, gratitude is an example of other-oriented socially-engaging emotion which are found to be felt more strongly by individuals from Asian cultures (like Japan), compared to European Americans [58]. A few other new dimensions generated in the present study were Meaning-making (M-M), Acceptance of Reality (AoR), and Self-care (SC). There is some conceptual similarity among some of these dimensions and dimensions/indicators from other existing well-being frameworks. For example, resilience is conceptually related to AoR and M-M [3]. ‘Meaningful Engagement’ (ME) is another dimension generated in the present study which has limited conceptual overlap with ‘Engagement’ and ‘Meaning’ [3,48,49]. Minor or routine activities or processes of ‘relating’ or ‘doing’ was found to represent ‘Meaningful engagement’ (merger of both meaning and engagement) in one of the Indian studies [59]. This conception is closer to the ME in the present study.

The dimension of ‘Self-acceptance & Respect included awareness of strengths, limitations as well as an emphasis on handling negative feedback and criticisms about self from others. Other existing frameworks that include similar dimensions namely, self-acceptance, self-esteem or self-respect [3,9,48]. Self-acceptance & Respect dimension in the present study emphasized elements of self in interaction with others and dignity under difficult situations, reflecting the complexity of the construct in Indian context and requires further investigation.

### 5.5. Dimensions of Positive Mental Health from Eudemonic and Hedonic Lens

The present study led to the development of some unique dimensions of positive mental health, as well as re-discovery of some dimensions which are well-documented in western well-being literature. The eight ‘new’ PMH dimensions that emerged from the Delphi-data were endorsed by majority (75% or more) of the panellists as relevant and they strongly suggested retaining Among these eight ‘new’ dimensions, ‘Emotional Regulation’ and ‘Self-reflection and Clarity’ were the two dimensions which were reported to be most relevant by the Delphipanelists. The rest of the dimensions were also reported to be relevant dimensions, endorsed by at least 3/4 of all the panelists. The emergent dimensions of PMH denoted convergences and divergences with existing literature. A few of the dimensions were found to be largely similar to the existing dimensions in western well-being literature (viz., Mastery & Competence, Positive Relations with others, Personal growth, Social contribution, etc.) suggesting their relevance in the contemporary Indian context However, finer/subtle differences in their meanings/constituents were observed between these dimensions and their western counterparts. A few new PMH dimensions emerged with no exact corresponding counterparts in western literature in models of well-being. Although there has been global research on these constructs, these have not been included as components of positive mental health in the available models of well- being, probably reflecting a heavy focus on Eurocentric conceptualizations of mental health.

Recent literature is rife with controversies relating to distinctions between eudaimonic and hedonic well-being. Eudaimonic well-being is primarily constituted by psychological well-being (psychologically functioning well) and social well-being (socially functioning well), whereas hedonic well-being is constituted by emotional well-being. Most of the dimensions generated in the present study clearly fall in the category of eudaimonic well-being, i.e., doing/functioning well in life. Only two dimensions (viz., Contentment & Gratitude and Emotional Regulation) could partially qualify as hedonic components of well-being by virtue of them being positive emotions (i.e., contentment experiences and savouring of positive emotions). The dimension of ‘Emotional Regulation’ though includes aspects of both positive and negative emotions; it does not exclusively fall under the category of hedonia as ‘regulation’ component is a core aspect of this dimension. In other words, ER is a combined dimension having both characteristics of hedonia and eudaimonia. Recent researchers have cautioned against the tendency to compare and contrast hedonia and eudaimonia so as to establish supremacy of one over other [60]. Rather, they have urged to pursue well-being studies in an integrated way as empirical research has found that eudaimonic and hedonic pursuits can exist in combination [61]. Social well-being, as defined by Keyes [10] is represented in the present study by only one dimension, viz., ‘Giving: Social contribution’. However, aspects of social well-being are also reflected in two more dimensions viz., Meaningful engagement and Positive relations with others. In both these cases, the context of well-being activities happens to be the larger community/society. However, conceptual fuzziness of self and others, especially in interdependent cultures like India, makes it difficult to distinguish between the realms of personal vs. social, thus raising questions on applicability of Keyes’ clear demarcations of psychological (personal) well-being and social well-being. The panellists, in the context of reporting criticalities of various dimensions, also underscored the inter-relationships of the dimensions- how they are related to each other and can potentially act in unison with each other.

### 5.6. Limitations, Future Directions and Implications

The study was exploratory in nature. Delphi experts’ responses regarding what would constitute positive mental health indicators are likely to have been influenced by personal as well as professional experiences and observations of clients in situations similar to the vignettes, ideas and beliefs based on Indian philosophical thoughts as well as contemporary cultural values. The data from second round of the Delphi survey were based on 30 out of the initial 35 respondents due to 5 drop-outs related to unexpected time constraints. One to one interviews with experts may have helped in clarifying the basis of their responses further. Exploration of lay experiences and perceptions of Indian adults related to these indicators and dimensions through one to one interviews and data triangulation in future studies can help in further validation of the positive mental health dimensions that emerged by using Delphi-methodology with experts.

There is a steadily growing body of evidence on the utility of mental health promotion and preventive measures with a call to broaden research attention beyond risk factors to include protective and promotive factors and to explore the public health benefits of this broadened approach [62,63,64,65]. In this context, contextually grounded conceptualisation of positive mental health in the present study can pave way for further research on development of ecologically valid assessment tools, cross cultural comparisons as well as designing of preventive and promotive intervention programs.

## Figures and Tables

**Table 1:** Basic socio-demographic details of the expert-participant sample (N = 35).

Basic Socio-Demographic Characteristics		Frequency	Percentage
**Age in years** *Range = 32–76 yrs.; Mean (SD) = 50.4 yrs.(11.04)*	Upto 50	14	40.0
	Above 50	21	60.0
**Gender**	Male	13	37.1
	Female	22	62.9
**Marital Status**	Unmarried	6	17.1
	Married	27	77.1
	Widow (er)	2	5.8

**Table 2:** Further details of expert-participant sample (N = 35).

Other Sample Characteristics		Frequency	Percentage
**Educational Qualification** *(highest degree)*	PhD/PsyD	20	57.1
	MD/MRPsych	6	17.1
	M. Phil	3	8.6
	Masters	5	14.3
	Bachelors	1	2.9
**Work Experience** *(in yrs.)*	5–7	3	8.6
	7–10	2	5.7
	11–15	5	14.3
	16–20	2	5.7
	>21	23	65.7
**Specialization** *(subject-area)*	Mental Health Professions *(Clin. Psych., PSW, Psych Nsg., Psychoanalysis, Coun. Psych.)*	26	74.3
	Behavioural Sc./Organizational Behaviour	3	8.6
	Well-being/Indian Psych.	2	5.7
	Other General *(e.g., Social, Gender, Human Development. & Family Studies)*	4	11.4
**Current work-setting**	Hospital	4	11.4
	Private practice	4	11.4
	Industry/Independent Counselling centre	3	8.6
	University/college	13	37.2
	Other/Multiple	10	28.6
**Primary work-responsibility**	Practice (Clinical, Counselling, Therapy & Coaching)	9	25.7
	Teaching/training	7	20.0
	Teaching, Research & Practice	8	22.8
	Other/different combinations	11	31.5

**Table 3:** Positive Mental Health indicators based on qualitative content analysis of responses in Delphi—Round 1.

PMH Indicator	Descriptions
**Dialogue for conciliation**	Conflict resolution through communications aimed at including significant others in arriving at an agreeable solution for oneself by decreasing resistance from others. It involves efforts (dialogues) at generating support from significant others whose support is not available on the issue at hand.
**Being flexible & Openness to multiple perspectives**	Openness in considering and exploring multiple options for handling situations, including new/unfamiliar options. It also includes being open to perspectives which are different from one’s own.
**Sense of Competence & Mastery**	Having a sense of competence and mastery in dealing with situations as well as developing skills to increase mastery
**Realistic appraisal of situation**	Actively exploring different sources of information and considering various elements of a situation to understand the given situation and the factors associated with it
**Finding creative solution**	Discovering unusual/original ways of handling a situation which maximizes benefits for all concerned and minimizes costs/bypasses constraints or barriers
**Proactive coping**	Anticipating potential difficulties in future and working on minimizing the same/consequences of the same, before they arise
**Balancing life domains**	Making active attempts to balance two/more domains of one’s life, when faced with challenging situations
**Motivating self**	Mobilizing one’s emotional energy to push oneself to work towards a valuable outcome (in the face of a difficult situation)
**Balancing short-term & long-term goals**	Prioritizing goals and setting action-plans
**Following norms**	Abiding by social norms/customs of the community (this was mentioned by experts in the context of facing a difficult situation and entailed giving up one’s valued goals when the same deviated from the norms prevalent in one’s culture)
**Applying one’s strengths**	Using one’s strengths in day-to-day life to handle various environmental demands
**Mobilizing support**	Being open to look out for and receive support to handle a difficult situation, exploring how to expand the usual sources of support (attempting to develop new support systems)
**Supporting others**	Supporting others in term of physical and emotional needs, sometimes despite having had a conflicting situation with the person (also includes motivating/inspiring others to work hard towards valued goals)
**Investing in relationships/connecting**	Connecting to others not for a specific purpose in a given context, but enjoying relationships and nurturing the same as intrinsically gratifying
**Tolerance/Empathy**	Tolerating others with whom one may have had disagreement. Empathizing with another person or her/his perspective (sometimes despite disagreement)
**Balancing needs of self and others**	Actively pay attention to others’ needs and not just one’s own, balance between needs of several members of the family, especially when the resources are limited
**Courage**	Acting on the basis of one’s convictions/beliefs and accepting the consequences of it, in contexts where there is a threat of disapproval or isolation/rejection/resistance from significant others/society in general. (It may be noted that ‘Courage’ emerged as a stance arrived at through internal clarity after attempts to dialogue have failed)
**Autonomous-connectedness**	Making decisions or acting on volitional basis rather than as a result of external pressures or internal compulsions, while at the same time actively making efforts to preserve or strengthen relationship with significant others (despite areas of disagreements)
**Accepting consequences of decisions/taking responsibility**	Being mentally prepared to handle consequences of what decision one makes/action one takes in a situation
**Internal clarity**	Taking time for self-reflection to become aware of one’s own wants, needs, motives, feelings, values, goals and the basis for the same and using this clarity to decide on a course of action
**Reviewing life’s goals and purpose**	Taking stock of what one has been doing, at critical points in life and reviewing what one wants to do in future, in the background of changing external realities
**Managing negative emotions**	Dealing with negative emotions which have surfaced in the context of difficulty, with the goal of maintaining external composure as well as internal calmness
**Non-impulsive responding**	Having frustration tolerance and being able to be non-impulsive in difficult situation
**Positive emotion in negative/challenging situations**	Being able to generate positive emotions and generate/maintain hope, during difficult situation
**Acceptance of reality**	Acceptance of the external reality, specifically the aspects of reality that cannot be changed
**Self-care**	Taking regular care of what one needs to do for one self for maintaining good physical and mental health and balanced life in general
**Self-acceptance/respect**	Accepting one’s self globally, respecting oneself with one’s mistakes/limitations and strengths
**Meaningful engagement**	Investing time in activities that are personally meaningful, expanding new sources of meaningful engagement when old sources are diminished
**Giving one’s best**	Giving the best that one can give in one’s regular/routine domain of work, without getting too preoccupied with the outcomes of it. This indicator was often mentioned in the context of a difficult situation
**Social contribution**	Doing something of value for people/community beyond the network of personal relationships. This has been coded when the nature of meaningful engagements involve contributing to a social cause, outside one’s routine domain of engagement.
**Meaning making**	Process of making sense of a negative situation/life event using one’s system of beliefs, values and philosophy, arriving at one’s own responses to distressing questions such as ‘why this?’, ‘why me?’.
**Contentment & gratitude**	Focusing on what one has within one self/in one’s life rather than being preoccupied with what may be lacking; Appreciating and deriving a sense of satisfaction from the good things in one’s life (sometimes despite being in a difficult situation) and experiencing a sense of gratitude
**Personal growth**	Intentions/efforts towards bringing about desired changes in personal attributes, patterns of feeling-thinking-behaving (mostly in the background of challenging situations)

**Table 4:** Positive Mental Health dimensions (Based on content analysis of responses in Delphi—Round 1).

	Broad Dimension *(with Number of Indicators Subsumed under It)*	Descriptions/Foundational Definition
**1**	**Mastery & Competence *(10)***	Efforts at managing demands in one’s life/environment for accomplishing valued outcomes and maintaining a overall sense of competence
**2**	**Positive relations with others *(6)***	Making efforts to develop and nurture relations with others through investing in relationships, providing support, having empathy, being open to seeking support and engaging in dialogues to resolve conflicts.
**3**	**Personal growth *(1)***	Intentions/efforts towards bringing about desired changes in personal attributes, patterns of feeling-thinking-behaving (mostly in the background of challenging situations)
**4**	**Self-acceptance/respect *(1)***	Accepting one’s self globally, respecting oneself with one’s mistakes/limitations and strengths
**5**	**Autonomy-Relatedness *(3)***	Making decisions or acting on volitional basis while actively nurturing relationships with significant others despite disagreements; Both elements (autonomy and relatedness) are seen as functioning conjointly, not one at the cost of other
**6**	**Self-reflection and clarity *(2)***	Arriving at a position of internal clarity through self-reflection and reviewing one’s life goals in the context of changing/challenging contexts, if needed
**7**	**Emotional regulation *(3)***	Regulating one’s own emotions through dealing with negative emotions, responding non-impulsively and generating positive emotions in the context of negative/challenging situations
**8**	**Acceptance of reality *(1)***	Acceptance of the external reality, specifically the aspects of reality that cannot be changed
**9**	**Self-care *(1)***	Taking regular care of what one needs to do for one self for maintaining good physical and mental health and balanced life in general
**10**	**Meaningful engagement *(1)***	Investing time in activities that are personally meaningful, expanding new sources of meaningful engagement when old sources are diminished
**11**	**Giving (Social contribution) *(2)***	‘Giving’ either in terms of giving one’s best to whatever one engages in one’s routine life, or contributing to society through taking on activities for a social cause
**12**	**Meaning making *(1)***	Process of making sense of a negative situation/life event using one’s system of beliefs, values and philosophy
**13**	**Contentment & gratitude *(1)***	Focusing on what one has within one self/in one’s life rather than being preoccupied with what may be lacking; Appreciating and deriving a sense of satisfaction from the good things in one’s life (sometimes despite being in a difficult situation) and experiencing a sense of gratitude

**Table 5:** Summary of Positive Mental Health Dimensions & Indicators emerged from Delphi-1 data.

PMH Indicator	Number of Experts Endorsing the PMHi (out of 35)	Number of Vignettes Generating the PMHi (out of 10)	Corresponding Dimensions
Dialogue for conciliation	31 (89%)	9	*Mastery & competence*
Being flexible & Openness to multiple perspectives	30 (86%)	8	
Sense of Competence & Mastery	29 (83%)	8	
Realistic appraisal of situation	27 (77%)	9	
Finding creative solution	24 (69%)	8	
Proactive coping	20 (57%)	9	
Balancing life domains	19 (54%)	5	
Motivating self	10 (29%)	8	
Balancing short-term & long-term goals	15 (43%)	8	
Following norms	4 (11%)	4	
Applying strengths	5 (14%)	4	
Dialogue for conciliation	31 (89%)	9	*Positive relations with others*
Mobilizing support	34 (97%)	10	
Supporting others	31 (89%)	5	
Investing in relationships/connecting	22 (63%)	8	
Tolerance/Empathy	16 (46%)	3	
Balancing needs of self and others	5 (14%)	3	
Personal Growth	15 (43%)	5	*Retained as Personal growth*
Self-Acceptance & respect	20 (57%)	7	*Retained as self-acceptance/respect*
Courage	23 (66%)	9	*Autonomy-* *Relatedness*
Autonomous-connectedness	18 (51%)	4	
Accepting consequences of decisions/taking responsibility	10 (29%)	4	
Internal clarity	22 (63%)	9	*Self-reflection and clarity*
Reviewing life’s goals and purpose	13 (37%)	6	
Managing negative emotions	21 (60%)	8	*Emotional regulation*
Non-impulsive responding	17 (49%)	9	
Positive emotion in negative/challenging situation	15 (43%)	10	
Acceptance of reality	34 (97%)	10	*Retained as acceptance of reality*
Self-care	28 (80%)	5	*Retained as self-care*
Meaningful engagement	23 (66%)	6	*Retained as meaningful engagement*
Giving one’s best	24 (69%)	6	*Giving (Social contribution)*
Social contribution	18 (51%)	5	
Meaning-making	14 (40%)	7	*Retained as meaning-making*
Contentment &gratitude	10 (29%)	7	*Retained as contentment & gratitude*

**Table 6:** Levels of agreement among expert-participants regarding match between PMH-indicators and Dimensions-Delphi Round2.

Dimensions	Indicators	Expert-Participants’ Responses (Percentage in Parenthesis) (N = 30)			
		Complete Agreement ^1^	Agreement with Suggestion/Comments ^2^	Disagreement and Requiring Modifying/Realigning ^3^	Complete Disagreement as PMH-i ^4^
**Mastery & Competence (M&C)**	**Dialogue for conciliation**	18 (60)	0	12 (40)	0
	**Being flexible &openness to multiple perspectives**	28 (93.3)	1 (3.3)	1 (3.3)	0
	**Sense of competence &mastery**	30 (100)	0	0	0
	**Realistic appraisal of situation**	26 (86.6)	4 (13.3)	0	0
	**Finding creative solution**	30 (100)	0	0	0
	**Proactive coping**	28 (93.3)	0	1 (3.3)	1 (3.3)
	**Balancing life domains**	28 (93.3)	1 (3.3)	1 (3.3)	0
	**Motivating self**	23 (76.6)	4 (13.3)	3 (10.0)	0
	**Balancing short-term & long-term goals**	27 (90)	1 (3.3)	2 (6.6)	0
	**Following norms**	12 (40)	5 (16.6)	7 (23.3)	6 (20)
	**Applying strengths**	29 (96.6)	0	1 (3.3)	0
**Positive Relations with others (PR)**	**Dialogue for conciliation**	30 (100)	0	0	0
	**Mobilizing support**	26 (86.6)	2 (6.6)	2 (6.6)	0
	**Supporting others**	28 (93.3)	1 (3.3)	1 (3.3)	0
	**Investing in relationships/connecting**	27 (90)	2 (6.6)	1 (3.3)	0
	**Tolerance/Empathy**	28 (93.3)	1 (3.3)	1 (3.3)	0
	**Balancing needs of self and others**	28 (93.3)	2 (6.6)	0	0
**Autonomy-Relatedness (A-R)**	**Courage**	15 (50)	4 (13.3)	11 (36.6)	0
	**Autonomous-connectedness**	30 (100)	0	0	0
	**Accepting consequences of decisions/taking responsibility**	25 (83.3)	5 (16.6)	0	0
**Self-reflection & Clarity (SR-C)**	**Internal clarity**	28 (93.3)	2 (6.6)	0	0
	**Reviewing life’s goals and purpose**	27 (90)	2 (6.6)	1 (3.3)	0
**Emotional Regulation (ER)**	**Managing negative emotions**	28 (93.3)	2 (6.6)	0	0
	**Non-impulsive responding**	28 (93.3)	1 (3.3)	1 (3.3)	0
	**Positive emotion in negative/challenging situation**	28 (93.3)	1 (3.3)	1 (3.3)	0
**Personal growth (PG)**	**Personal growth (PG)**	28 (93.3)	0	2 (66)	0
**Self-acceptance/respect (SA-R)**	**Self-acceptance/respect (SA-R)**	29 (96.6)	0	1 (3.3)	0
**Acceptance of reality (AoR)**	**Acceptance of reality (AoR)**	28 (93.3)	1 (3.3)	1 (3.3)	0
**Self-care (SC)**	**Self-care (SC)**	29 (96.6)	1 (3.3)	0	0
**Meaningful engagement (ME)**	**Meaningful engagement (ME)**	24 (80)	2 (6.6)	4 (13.3)	0
**Giving: Soc. Contri. (G-SC)**	**Giving one’s best**	23 (76.6)	3 (10)	4 (13.3)	0
	**Social contribution**	27 (90)	2 (6.6)	1 (3.3)	0
**Meaning-making (M-M)**	**Meaning-making (M-M)**	25 (83.3)	2 (6.6)	2 (6.6)	1 (3.3)
**Contentment & gratitude (C&G)**	**Contentment & gratitude (C&G)**	24 (80)	3 (10)	3 (10)	0

^1^ It denotes panelist agreeing fully with how the indicators are grouped under the dimensions in the present study by the researchers; ^2^ It denotes panelist’s fairly high degree of agreement with how the indicators are grouped under the dimensions in the present study. However, unlike complete agreement where the panelist had nothing to add/comment upon, here s/he suggests other options of grouping over and above the existing one, or some changes in terminology. ^3^ The panelist, in this case, disagrees with researchers in the way the indicators are grouped under the dimensions or their definitions and, in many cases, suggests alternatives which, according to him/her, suits better.^4^ In this case, there is a complete and emphatic disagreement with the researchers about not only the grouping, but also the legitimacy of the indicator itself in the context of PMH.

**Table 7:** Frequency of endorsement of Relevance of New PMH-Dimensions by Delphi Round 2 panelists (N = 30).

Proposed New PMH Dimension	Endorsement/Non-Endorsement			Final Decision for Present Study (%)
	Relevant (%)	Relevant with Modification/Re-Alignment (%)	Non-Relevant (%)	
**Emotional Regulation**	27 (90)	2 (6.6)	1 (3.3)	Retained
**Self-reflection & Clarity**	27 (90)	1 (3.3)	2 (6.6)	Retained
**Meaning-making**	25 (83.3)	2 (6.6)	3 (10)	Retained
**Acceptance of reality**	25 (83.3)	1 (3.3)	4 (13.3)	Retained
**Contentment & gratitude**	25 (83.3)	1 (3.3)	4 (13.3)	Retained
**Self-care**	25 (83.3)	1 (3.3)	4 (13.3)	Retained
**Giving: Soc. Contri.**	24 (80)	2 (6.6)	4 (13.3)	Retained
**Meaningful engagement**	23 (76.6)	1 (3.3)	6 (20)	Retained

**Table 8:** Frequency of endorsement of criticality of PMH-dimensions by Delphi Round 2 panellists (N = 25).

List of PMH-Dimensions	Endorsements as Top-5	
	Number of Expert-Panelists	Percentage
**Mastery & Competence (M&C)**	18	72
**Emotional regulation (ER)**	17	68
**Self-acceptance/respect (SA-R)**	14	56
**Positive relations with others (PR)**	13	52
**Personal growth (PG)**	11	44
**Self-reflection and clarity (SR-C)**	11	44
**Acceptance of reality (AoR)**	11	44
**Contentment & gratitude (C&G)**	9	36
**Self-care (SC)**	8	32
**Giving (social contribution) (G-SC)**	8	32
**Autonomy-Relatedness (A-R)**	6	24
**Meaning-making (M-M)**	6	24
**Meaningful engagement (ME)**	6	24

Data from five panellists of Delphi Round2 were not available as they did not respond to this specific query in the survey-proforma.
